# From Cytokines to Biomarkers: Mapping the Immunopathology of Inflammatory Bowel Disease

**DOI:** 10.3390/cells14201589

**Published:** 2025-10-13

**Authors:** Sarah Baum, Kamron Hamedi, Caroline Loftus, Gannett Loftus, Emily-Rose Zhou, Sergio Arce

**Affiliations:** 1 Anesthesiology Residency, University of Virginia Health, University of Virginia Hospital, Charlottesville, VA 22908, USA; thesarahbaum@gmail.com; 2 School of Medicine Greenville, University of South Carolina, Greenville, SC 29605, USA; sarceusc@gmail.com; 3 Internal Medicine Residency, Virginia Mason Franciscan Health, St. Joseph Medical Center, Tacoma, WA 98405, USA; 4 School of Medicine Columbia, University of South Carolina, Columbia, SC 29209, USA; caroline.loftus@uscmed.sc.edu (C.L.); gannett.loftus@uscmed.sc.edu (G.L.); 5 Internal Medicine Residency, Yale School of Medicine, Yale New Haven Hospital, New Haven, CT 06510, USA; erzprov35@gmail.com; 6 Prisma Health Cancer Institute, Prisma Health System, University of South Carolina, Greenville, SC 29605, USA

**Keywords:** IBD, cytokines, biomarkers, TNF, intestinal epithelial barrier

## Abstract

Inflammatory bowel disease (IBD) is a chronic immune-mediated condition of the gastrointestinal tract, characterized by dysregulated inflammatory responses throughout the gastrointestinal tract. It includes two major phenotypes, Crohn’s disease (CD) and ulcerative colitis (UC), which present with varying gastrointestinal and systemic symptoms. The pathophysiology of IBD is multifactorial including genetic predisposition, mucosal and epithelial dysfunction, environmental injury, and both innate and adaptive immune response abnormalities. Several predisposing genetic factors have been associated with IBD explaining the strong hereditary risk for both CD and UC. For example, Caspase Recruitment Domain 9 (CARD9) variant rs10781499 increases risk for IBD, while other variants are specific to either CD or UC. CD is related to loss-of-function mutations in the nucleotide oligomerization domain containing the protein 2 (NOD2) gene and Autophagy-Related 16-like 1 (ATG16L1) gene. UC risk is increased particularly in Chinese populations by the A-1661G polymorphism of the Cytotoxic T-lymphocyte antigen 4 (CTLA-4) gene. This abnormal CTLA-4 interferes with B- and T-cell responses causing predisposition to autoimmune conditions. Previous studies suggested that IBD results from breakdown of the adaptive immune system, primarily of T-cells. However, new evidence suggests that a primary breakdown of the innate immune system in both CD and UC increases susceptibility to invasion by viruses and bacteria, with a compensatory overactivation of the adaptive immune system as a result. When this viral and microbial invasion continues, further damage is incurred, resulting in a downward cycle of further cytokine activation and epithelial damage. Released biomarkers also affect the permeability of the epithelial membrane, including lactoferrin, nitric oxide (NO), myeloperoxidase (MPO) and its activation of hypochlorous acid, matrix metalloproteinases (MMPs), especially MMP-9, omentin-1, and others. Increased macrophage and dendritic cell dysfunction, increased neutrophil activity, increased numbers of innate lymphoid cells, increased T-cells with decreased regulatory T-cells (Tregs), and changes in B-cell populations and immunoglobulin (Ig) functions are all associated with IBD. Finally, treatment of IBD has typically consisted of medical management (e.g., aminosalicylates and corticosteroids) and lifestyle modification, and surgical intervention in extreme cases. New classes of medications with more favorable side effect profiles include anti-integrin antibodies, vedolizumab, etrolizumab, and carotegrast methyl. Additionally, fecal microbiota transplant (FMT) is a newer area of research for treatment of IBD along with TNF-blockers, JAK inhibitors, and S1PR modulators. However, expense and long preparation time have limited the usefulness of FMT.

## 1. Introduction

Under normal conditions, the GI immune system is an intricate network of physical barriers, innate immune cells, and adaptive immune cells that constantly monitor the gut environment and respond to potential pathogens. When these response networks are compromised, diseases like IBD can ensue. In a healthy gut, responses initiate from baseline anatomical defenses including the epithelium of the gut, crypts of Lieberkühn, lamina propria, Peyer’s patches (PPs) with gut-associated lymphoid tissue (GALT), and mast cells throughout the mucosa. The GI epithelium must be able to distinguish between the commensal microbiome (those bacteria that the host is dependent on for survival) and the microbiota that is harmful to the host and necessitates an immune response. The epithelium contains enterocytes, goblet cells, Paneth cells (crypts of Lieberkühn only), chemosensory tuft cells, and M-cells (over Peyer’s patches), which each contribute to maintaining a healthy gut by responding appropriately when there is dysfunction of the normal crosstalk between the microbiota and the intestinal epithelial cells (IECs). Paneth cells secrete antimicrobial peptides like α-defensins, goblet cells produce mucus to limit microbial contact, and tuft cells sense parasitic infections through IL-25 signaling. M-cells facilitate antigen sampling and presentation in PP, supporting immune surveillance. These cellular defenses are supported by the crypts of Lieberkühn, which house intestinal stem cells that differentiate into various epithelial lineages under Wnt signaling. Additionally, in response to cytokines such as interleukin (IL)-22 and interferon gamma (IFNγ), goblet cells exocytose mucin into the GI lumen to protect the epithelium from direct pathogen contact. Paneth cell dysfunction or impaired stem cell renewal within these crypts has been linked to the development of CD.

Beyond the epithelium, the lamina propria, GALT, and resident immune cells provide additional immune regulation. Macrophages and dendritic cells (DCs) in the lamina propria contribute to antigen presentation and tolerance. In IBD, this balance is disrupted, often favoring pro-inflammatory pathways. Macrophages shift toward a macrophage type 1 (M1) phenotype, producing cytokines via toll-like receptor (TLR) activation, while DCs promote the differentiation of T helper 1 (TH1) and 17 (TH17) cells. B-cell activation in GALT results in immunoglobulin class switching and the production of IgA, IgG, and IgE. Mast cells further contribute by releasing inflammatory mediators such as tumor necrosis factor alpha (TNF-α) and histamine, which recruit neutrophils and amplify local inflammation.

When these protective mechanisms fail, persistent immune activation against the gut microbiota ensues. This leads to the chronic mucosal and transmural inflammation characteristic of UC and CD, respectively. The specific immune pathways involved differ between the two diseases. UC is restricted to the colon and involves superficial mucosal inflammation driven by TH17 and TH2 cells, often accompanied by impaired mucus production and increased epithelial permeability. In contrast, CD can affect any part of the GI tract and is marked by deeper, transmural inflammation, granuloma formation, and a prominent TH1 and TH17 cytokine profile. Deficiencies in autophagy and antimicrobial peptide production further contribute to disease progression in CD.

IBD incidence continues to rise globally, particularly in developed countries, with the highest prevalence in the United States [1]. While genetic susceptibility, environmental factors, and microbial imbalances all contribute, the central mechanism involves failure of the intestinal immune system to regulate its response to normally non-pathogenic stimuli [2]. This breakdown in tolerance leads to excessive inflammation, tissue damage, and fluctuating periods of disease relapse and remission [2,3]. Early recognition of disease subtype and immune dysregulation is crucial for diagnosis, targeted therapy, and long-term management.

This review describes the intricate protective network of the GI immune system and how its dysfunction leads to the development of IBD. This network is described by cell type, highlighting the cytokines produced by each cell type with their functions and involvement in IBD. Clinically relevant biomarkers with current or potential use for disease diagnosis, differentiation, treatment responsiveness, and surveillance will also be discussed in the context of the previously described disease process. These biomarkers include inflammatory cytokines, calprotectin, lactoferrin, NO, MPO, omentin-1, MMP-9, C-reactive protein (CRP), and exosomal component microRNA (miR)-223 [4,5,6]. This review will present a broad overview of the cytokine pathways, biomarkers, and their related treatments to help connect molecular biology and pathophysiology with clinical practice.

## 2. Normal and Disease Pathophysiology of the Gastrointestinal Immune System in IBD

### 2.1. Mucous Layers and the Epithelial Barrier

**Figure 1:**
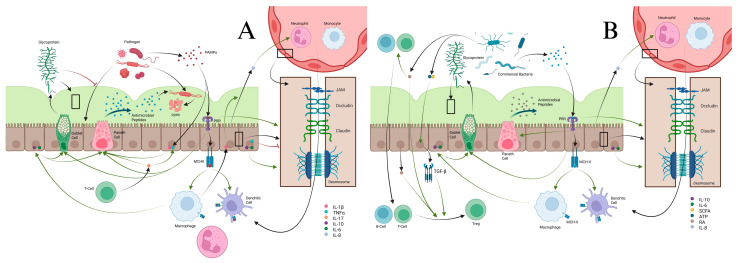
(**A**) Pathogen interactions with epithelial barriers stimulates a host of cytokine responses through various pathways. Pathogens release PAMPs, which are released and recognized by PRRs located on epithelial cells. These cause expression of MHCII by epithelial cells for antigen presentation to macrophages and dendritic cells. Activated macrophages release IL-6, IL-10, and IL-1β. IL-6 and IL-10 stimulate goblet cells to produce more glycoprotein to strengthen and maintain the mucus layer, increase antimicrobial peptide production from Paneth cells, and strengthen the epithelial barrier. PRR activation itself also stimulates glycoprotein production, increases Paneth cell activity, and tightens the epithelial barrier. Mucus itself prevents pathogen migration across the epithelial barrier and restrains them for destruction by anti-microbial peptides. IL-17 from T-cells increases Paneth cell activity and increases epithelial cell production of IL-1β and TNF-α. IL-8 naturally produced by epithelial cells, and increased under the influence of IL-1β from epithelial cells and macrophages, increases migration of neutrophils and macrophages from blood vessels into the lamina propria. TNF-α helps weaken the epithelial barrier to help with immune cell migration into the lamina propria. (**B**) The epithelial barrier has important roles in preserving homeostasis. In homeostasis, PAMPs from commensal bacteria stimulate maintenance of the mucus membrane and antimicrobial peptides from goblet and Paneth cells. The PRRs recognizing these commensal PAMPs lead to anergy of macrophages and dendritic cells. Commensal bacterial produce ATP and SCFA, which help recruit resident B-cells and T-cells and stimulate TGF-β production from epithelial cells. RA and TGF-β from epithelial cells cause differentiation of T-cells to Tregs, which promote tolerance of commensal bacteria.

The first mechanism in gut homeostasis is the mucous layer and its associated epithelial barrier. This layer is primarily produced by goblet cells, which secrete mucin glycoproteins, the major structural component of mucus [7]. The mucous layer prevents bacterial invasion both by physically blocking adhesion to epithelial cells and by delivering antimicrobial peptides. Among these are cathelicidins and α-defensins secreted by Paneth cells, which disrupt bacterial membranes and trap invading pathogens [8,9]. If bacteria bypass this defense, then they encounter the epithelial barrier composed of several cell types, including goblet, Paneth, and epithelial cells, which maintain selective permeability [8]. Integrity is sustained through tight junctions (claudins, occludins, junctional adhesion molecules) and desmosomes. Pattern recognition receptors (PRRs) identify pathogen-associated molecular patterns (PAMPs) and trigger antimicrobial peptide and mucin production while also presenting antigens via major histocompatibility complex (MHC)-II to macrophages and DCs [9,10]. Cytokines such as IL-10 and IL-6 from macrophages enhance mucin secretion, epithelial proliferation, and barrier reinforcement, balancing tolerance to commensal bacteria with protection against pathogens [11,12]. Commensal bacteria produce metabolites including short-chain fatty acids (SCFAs), adenosine triphosphate (ATP), and retinoic acid (RA), which further promote tolerance by stimulating transforming growth factor beta (TGF-β) production and Treg differentiation [13,14]. Pro-inflammatory cytokines produced in response to pathogens such as IL-1β, TNF-α, and IL-17 regulate antimicrobial peptide production, increase epithelial permeability, and drive immune cell recruitment [12,15,16,17,18,19,20]. IL-17A and IL-17F specifically increase TNF-α production in IECs, increasing barrier permeability for immune cell recruitment [12,16]. IL-8 is produced in small amounts by the intestinal epithelium to attract resident macrophages and neutrophils [17]. During inflammatory reactions, IL-1β released from epithelial cells and macrophages increase production of IL-8, signaling downstream neutrophil and macrophage recruitment (Figure 1) [18]. Activated neutrophils shed soluble IL-6R (sIL-6R), which binds with IL-6 and is recognized by the epithelial cell receptor gp130, causing production of monocyte chemoattractant protein-1 (MCP-1), also known as chemokine (C-C motif) ligand 2 (CCL2) [9,17]. Detection of bacterial antigens by epithelial PRRs also causes the release of C-X-C motif chemokine 5 (CXCL5), influencing macrophages to increase TNF-α production [19,20].

In UC, thinning and erosion of the mucous layer may occur before and after inflammation, establishing a cycle of barrier loss and bacterial invasion [11,21]. Genetic influences include reduced MUC2 expression, mutations in MUC2, MUC3, and MUC19, and impaired goblet cell differentiation mediated by atonal homolog 1 (ATOH1) and Krüppel-like factor (KLF-4) [22,23,24,25,26]. Chronic inflammation disrupts mucin glycosylation, creating unstable glycans that degrade rapidly; Core 1 β3-Galactosyltransferase-Specific Molecular Chaperone (COSMC) mutations further link defective glycosylation to UC pathogenesis [27,28]. During active UC, goblet cell density, mucin secretion, and differentiation markers decline, but these changes often normalize during remission [24,25,26].

CD, in contrast, is frequently characterized by normal or increased mucous production, but pathogen clearance is impaired, often due to Paneth cell dysfunction. Mutations in autophagy-related genes (ATG5, ATG16, IRGM1) reduce antimicrobial exocytosis, while NOD2 mutations further impair antimicrobial secretion, increasing susceptibility [29,30,31]. Both UC and CD also demonstrate mucin glycosylation defects secondary to chronic inflammation, further compromising barrier stability.

Microbiome changes contribute significantly to barrier dysfunction. In UC, sulfide-producing bacteria such as Desulfovibrio destabilize mucin disulfide bonds, while mucolytic species including *Ruminococcus torques*, *Bacteroides fragilis*, and *E. coli* accelerate mucin degradation [24,32,33]. CD exhibits greater microbiome instability, with loss of beneficial commensals such as Faecalibacterium, Methanobrevibacter, and Anaerostipes, alongside expansion of pathogenic species including *E. coli*, Fusobacterium, and Collinsella, some of which may distinguish CD from UC [34,35]. Loss of commensals producing indole derivatives reduces aryl hydrocarbon receptor (AhR)-mediated anti-inflammatory signaling and Treg differentiation, further promoting inflammation [36,37].

Epithelial dysfunction is also an important aspect in the development of both UC and CD. As more invasive bacteria colonize the gut and the mucous barrier is damaged, bacterial components more frequently reach the epithelial barrier and initiate the previously discussed inflammatory cytokine pathways. This increased inflammation, particularly when prolonged, leads to epithelial damage [38]. Cytokines, including IL-2, IL-6, IL-8, TNF-α, and IFNγ, increase tight junction permeability through various means, including recruitment of neutrophils, monocytes, and increasing TH1 activity [39]. IL-10 knockout mice, which represent a loss of tolerance to commensal bacteria, epithelial repair, and inflammation attenuation, showed normal barrier function until inflammation was initiated by contact with bacteria. Similar deficiencies seen in humans have resulted in the development of IBD, demonstrating the effect inappropriate inflammation has on epithelial barrier function [40]. Inflammation leads to structural changes in claudins (which compose the tight junctions), leading to increased barrier permeability in CD and UC [22]. This is further demonstrated by claudin-7 knockout mice who demonstrated increased paracellular flux, increased tight junction permeability, and development of colitis [39]. TNF-α particularly causes increased production of claudin-2, which increases paracellular flux and causes the endocytosis of claudin-5 and claudin-8, weakening the barrier. Increased IFNγ, of which IL-2 increases production through TH1 activity, also causes downregulation of various claudins and occludins needed for proper tight junction function [41]. CD epithelial cells also failed to produce thymic stromal lymphopoietin (TSLP), an important cytokine in the IL-12 family. TSLP interacts with DCs to cause TH2 differentiation, which can help diminish IL-12 and IFNγ from TH1 cells. Loss of this cytokine leads to decreased regulation of inflammation and increased tight junction permeability from IFNγ activity [41]. Mutation of the TLR-4 gene leads to decreased PRR recognition of Gram-negative lipopolysaccharides (LPSs) [38]. This decreased recognition allows for colonization by Gram-negative bacteria like Vibrio cholerae to produce zonula occludens toxin (ZOT), a virulence factor that increases diarrhea by breaking up the interlinking strands that comprise epithelial tight junctions [42]. These various sources of epithelial damage lead to increased IL-13 production from innate lymphoid cell (ILC) 2 and TH2 cells to promote epithelial repair. However, excessive IL-13, which has been observed in UC tissue, causes increased paracellular permeability, most likely due to increased claudin-2 production [38]. Excessive epithelial apoptosis has also been noted in UC and CD and is primarily mediated by TNF-α. While part of the normal gut immune response, excessive stimulation leads to overproduction of TNF-α and increased apoptosis [43]. One mechanism for this apoptosis also intersects with mutations in NOD2. TNF-α increases IL-32 production in IECs, a cytokine involved in apoptotic signaling pathways. NOD2 mutations also increase IL-32 production, leading to increased apoptosis. IL-32 released into the inflammatory milieu can then increase TNF-α activity, forming a positive feedback loop [16].

Multiple biomarkers influence epithelial integrity. Lactoferrin maintains microbiota diversity, lyses bacteria, and upregulates tight junction proteins including claudin-1, occludin, and zonula occludens (ZO)-1. A reduction in this biomarker is associated with the development of both forms of IBD [44,45,46,47]. Lactoferrin supports the growth of bacteria with low iron requirements, including Bifidobacteria and Lactobacillus, which comprise a major part of the intestinal microbiome and improve the integrity of the intestinal barrier [45,46]. Lactoferrin exhibits antimicrobial activity by sequestration of iron, thus promoting an iron-deficient environment, and by cellular lysis of both Gram-positive and Gram-negative bacteria, including opportunistic strains of *Escherichia coli*, *Bacillus subtilis*, *Shigella dysenteriae*, *Salmonella typhimurium*, *Streptococcus* spp., *Enterococcus* spp., and *Staphylococcus* spp. [44,45].

Exosomal factors also modulate repair and inflammation: miR-223 promotes pro-inflammatory signaling, whereas annexin A1 and miR-21 enhance epithelial repair [48,49,50]. miR-223 promotes a pro-inflammatory response by activating the IL-23/TH17 pathway, leading to downregulation of claudin-8 and weakening the tight junctions of the intestinal epithelial barrier [48]. In contrast, annexin A1, a phospholipid-binding protein released by neutrophils by means of exosomes or by shedding of the plasma membrane, has been demonstrated to induce IEC migration and wound repair. Annexin A1 accomplishes this by activating formyl peptide receptor 1, a receptor involved in chemotaxis and generation of high levels of reactive oxygen species (ROS), leading to microbial death and consequent wound repair. Formyl peptide receptor 1 activates the nicotinamide adenine dinucleotide phosphate (NADPH) oxidase 1 (NOX1), an enzyme expressed on IECs that generates ROS [49]. Similarly, miR-21, an miRNA synthesized in colonic epithelial cells, promotes colonic IEC proliferation and migration. miR-21 is induced by the binding of substance P, a neuropeptide secreted by intestinal macrophages, neurons, and endocrine cells, to neurokinin-1 receptor (NK1R), a receptor located on IECs and immune cells within the intestinal mucosa [50].

NO reacts with superoxide within the inflamed intestine to form peroxynitrite, which induces peroxidation of the membrane lipids and damages DNA by activation of poly(ADP-ribose) synthetase (PARS), a nuclear enzyme, resulting in a reduction in intracellular stores of ATP and NAD+ and consequently damaging the tight junctions of the mucosal barrier [51,52]. NO also prevents DNA synthesis by inhibition of ribonucleotide reductase [53]. NO may also induce dysfunction of the mitochondrial respiratory complex, further promoting intestinal injury [54]. NO is significantly elevated in active CD and active UC, indicating its potential as a biomarker for IBD [52,55,56]. One study demonstrated increased activity of NO synthase in both active UC and active CD, although the activity of NO synthase in patients with UC was two-fold higher than the activity of NO synthase in patients with CD despite a similar degree of inflammation upon histology [57]. Another study showed similar increases in inducible NO synthase (iNOS) activity in the inflamed colonic mucosa of patients with UC and CD [56].

Like NO, MPO, an enzyme stored in neutrophil granules that induces production of ROS, promotes breakdown of the mucosal barrier. IL-8 induces MPO degranulation in neutrophils, leading to generation of hypochlorous acid and tyrosyl radicals within the phagosome [58,59]. Hypochlorous acid inhibits activity of glyceraldehyde-3-phosphatase in colonic epithelial crypt cells, leading to apoptosis, increasing mucosal permeability. Additionally, hypochlorous acid inhibits the activity of protein tyrosine phosphatases by oxidation, leading to increased activity of p38a and extracellular signal regulated kinase (ERK1/2), which are both mitogen-activated protein kinases (MAPKs) that transform extracellular signals into cellular effects [59]. P38a and ERK1/2 promote generation of IL-8 in colonic epithelial cells [60]. P38 stabilizes TNF-α mRNA, promoting expression and production of inflammatory cytokines like IL-1 and IL-6 through positive feedback in mucosal macrophages [59,61]. Hypochlorous acid inactivates phosphatase and tensin homolog (PTEN), a tumor suppressor protein that prevents the conversion of phosphatidylinositol 4,5-bisphosphate (PIP2) to phosphatidylinositol 3,4,5-trisphosphate (PIP3). With PTEN inactivated, increased PIP3 leads to increased nuclear factor kappa-light-chain-enhancer of activated B-cells (NF-κB) [59]. However, hypochlorous acid may also inhibit NF-κB, providing a measure of mediation for its pro-inflammatory effects. Hypochlorous acid generates chloroamines, which inactivate the inhibitor of NF-κB kinase and repress NF-κB [62]. Multiple studies have demonstrated an increase in fecal MPO in patients with active UC and CD [63,64,65]. Fecal MPO has also been shown to have similar precision to fecal calprotectin in the prediction of endoscopic disease activity in moderate to severely active IBD. Elevated levels of MPO at baseline predict a more complicated course of disease, such as relapses, requirement of corticosteroids or escalation of biologic therapies, hospitalization related to IBD, and surgery. Fecal MPO levels are found to then decrease in IBD patients upon treatment with biologics [64].

Omentin-1, a cytokine expressed in visceral adipocytes, supports the integrity of the mucosal barrier by reducing endoplasmic reticulum (ER) stress and by upregulating tight junction proteins [66]. ER stress may play a significant role in UC by promoting dysfunction of the intestinal barrier, epithelial cell death, and intestinal inflammatory responses through the NF-κB pathway [67]. By decreasing ER stress, omentin-1 inhibits apoptosis via anti-apoptotic protein B-cell lymphoma 2 (BCL-2), which inhibits the permeabilization of the mitochondrial outer membrane and decreases pro-apoptotic protein Bax [68,69]. Omentin-1 is also expressed in intestinal Paneth cells and aids in defense against invasion by pathogenic bacteria [70]. Unlike biomarkers such as CRP and TNF-α, omentin-1 has been shown to be negatively correlated with disease activity in CD as well as UC [71,72].

MMP-9 is produced by macrophages, neutrophils, and fibroblasts and amplifies the immune response in diseases involving chronic inflammation [73]. It disrupts the integrity of the mucosal barrier by degrading tight junction proteins and collagens in the basement membrane and extracellular matrix (ECM) [74,75]. MMP-9 then increases inflammation by promoting diffusion of bacterial LPS across the mucosal barrier and diffusion of neutrophil granule components including MMPs and calprotectin into the lumen of the intestine [74,75]. MMP-9 also inhibits goblet cell differentiation by activating Notch-1, which decreases the production of mucin [26]. Fecal MMP-9 levels are significantly elevated in patients with UC as compared to patients without IBD [76]. Fecal MMP-9 levels significantly correlate with endoscopic, histological, and clinical activity in UC, and are highly increased in patients with mucosal erosions and mucosal ulcerations [76,77]. As compared to fecal calprotectin, fecal MMP-9 has been demonstrated to be more strongly correlated with disease activity in UC [77]. Additionally, serum MMP-9 levels are significantly elevated in CD patients who relapse within 2 years as compared to non-relapsing CD patients, thus indicating that serum MMP-9 may be a valuable biomarker in predicting flares in CD as well [78].

### 2.2. Macrophages/Monocytes

**Figure 2:**
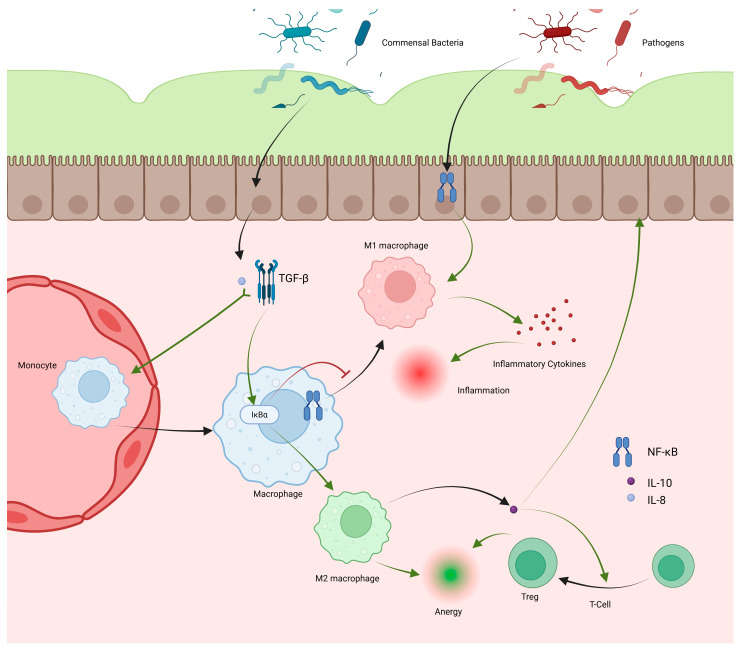
Macrophages hold important roles in promoting tolerance to commensal bacteria and inciting inflammation against pathogens. Macrophages are recruited as circulating monocytes, which migrate into the lamina propria, becoming macrophages. IL-8 and TGF-β produced by epithelial cells under the influence of commensal bacteria increase monocyte recruitment and increase IκBα activity in macrophages. IκBα inhibits the NF-κB pathway leading to decreased M1 differentiation and increased M2 differentiation. M2 macrophages promote anergy and produce IL-10, which promotes Treg differentiation, further promoting anergy. NF-κB induced by pathogens interacting with epithelial cells promotes M1 differentiation, leading to cytokine release and inflammation.

Bacteria that bypass the mucous and epithelial barriers encounter resident macrophages in the GI lamina propria. These cells originate from bone marrow-derived monocytes that continuously migrate into the GI tract. Recruitment is mediated by IL-8 and TGF-β secreted by IECs in response to commensal bacteria (Figure 2) [13,79]. TGF-β induces mothers against decapentaplegic homolog (SMAD) transcription factors, which stabilize inhibitor kappa B alpha (IκBα) by preventing its degradation and inhibit the NF-κB pathway. As NF-κB promotes pro-inflammatory cytokine production, this inhibition renders resident macrophages anergic to commensals (Figure 2). These anergic macrophages produce IL-10, maintaining the mucosal barrier and promoting Treg differentiation (Figure 2) [79].

When bacteria contact epithelial cells, CCL2 is released and binds C-C motif chemokine receptor 2 (CCR2) receptors [19]. CCR2 signaling recruits circulating monocytes to the small intestine and colon, where they differentiate into resident macrophages or, under inflammatory conditions, into CD14highHLA-DRlowCD163lowCD64+ macrophages. These produce IL-1, IL-6, IL-23, and TNF-α and display respiratory burst activity (Figure 2). IL-23, a member of the IL-12 family, drives TH1 and TH17 responses, both crucial in IBD pathology [79,80,81]. TNF-α disrupts barrier function by altering tight junctions and maintaining inflammation. Thus, macrophage cytokine activity is central to the development of UC and CD [10,82].

**Figure 3:**
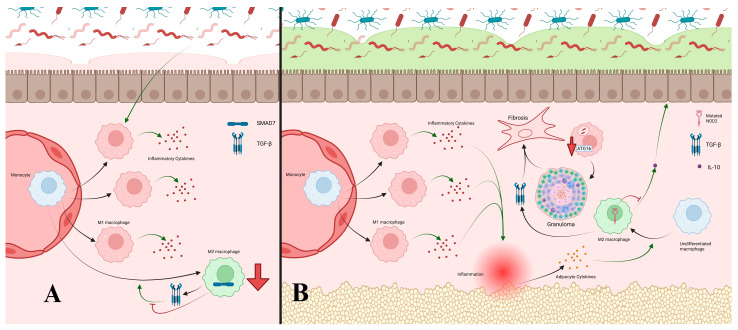
(**A**) Increased PAMP exposure in UC from weakened barriers leads to increased monocyte recruitment and differentiation into M1 macrophages due to the NF-κB pathway. This also results in decreased M2 differentiation, particularly through increased SMAD7 activity resulting in inhibition of the TGF-β pathway, further shifting macrophage differentiation to M1. M1 cells produce inflammatory cytokines and perpetuate inflammatory responses, further damaging defensive barriers. (**B**) In CD, increased exposure to PAMPs leads to accelerated monocyte recruitment and differentiation to M1 macrophages similar to the previously discussed pathways. Continued inflammation results in irritation of adipocytes and release of adipokines like adiponectin, which promote differentiation of macrophages to M2 macrophages. However, mutated NOD2 associated with CD impairs release of anti-inflammatory IL-10, limiting the anti-inflammatory effects of M2 cells. Mutations in ATG16 causing impaired autophagy prevent effective clearance of bacteria by M1 cells, leading to persistent inflammation and eventual granuloma formation. This granulation, along with TGF-β from M2 macrophages, form areas of fibrosis in the gastrointestinal tract.

Macrophages play a critical role in UC progression. As bacterial translocation increases through weakened barriers, macrophages recognize bacterial products, produce cytokines, and activate DCs and T-cells. Chronic exposure favors differentiation of recruited monocytes into pro-inflammatory M1 macrophages, sustaining cytokine production (Figure 3). During resolution, TGF-β and IL-10 promote anti-inflammatory macrophage type 2 (M2) polarization [4,83]. However, overexpression of SMAD7, an inhibitor of TGF-β, reduces M2 differentiation in UC, prolonging M1 dominance and epithelial damage (Figure 3) [84]. Commensals such as Clostridium butyricum promote M2 polarization, and their loss exacerbates UC inflammation, suggesting therapeutic potential in enhancing M2 differentiation [85].

CD exhibits broader macrophage dysfunction. Like UC, M1 activity is elevated by chronic inflammation, but CD also displays increased M2 macrophages, which contribute to healing and fibrosis [4,83,86]. The mechanisms remain unclear, but prolonged macrophage-driven inflammation with limited neutrophilic response may underlie CD’s transmural lesions [87]. Cytokines from mesenteric fat, exposed through these lesions, promote M2 polarization, explaining their abundance in CD (Figure 3) [88]. NOD2 mutations inhibit IL-10 production by M2 macrophages, impairing anti-inflammatory regulation (Figure 3) [4,89]. CD macrophages also exhibit defective bacterial clearance, leading to granuloma formation. Impaired cytokine secretion, degraded within macrophages, reduces neutrophil recruitment and bacterial elimination. This was demonstrated in CD macrophages failing to clear phagocytosed *E. coli*. Mutations in NOD2, IL-23 receptor subunits, and ATG16L1 impair autophagy and pathogen degradation, contributing to granuloma development, similar to tuberculosis (Figure 3) [4,90,91]. TGF-β from M2 macrophages further drives fibrosis, producing granulation and fibrotic tissue characteristic of CD (Figure 3) [4,86,92].

Several biomarkers influence macrophage differentiation. CRP promotes M2 polarization in its soluble pentameric form, but under inflammation it dissociates into a monomeric isoform that induces M1 differentiation and IL-1β, IL-6, and TNF-α release [93]. The monomeric isoform, insoluble in plasma, accumulates in inflamed intestinal tissue, amplifying local inflammation [94]. CRP correlates more strongly with CD than UC, likely due to higher IL-6 levels and transmural inflammation in CD [95,96]. However, ~15% of healthy individuals show no CRP rise during inflammation. A serum CRP > 5 mg/dL is highly specific but poorly sensitive for predicting endoscopic activity in IBD [97]. With a half-life of 19 h, CRP responds rapidly to acute inflammation, making it a useful marker of disease activity [98]. CRP is produced by hepatocytes primarily in response to IL-6, with IL-1β and TNF-α providing synergistic stimulation [99,100]. IL-6 also drives CRP production in adipocytes, and in CD, translocated gut bacteria and mesenteric fat hyperplasia amplify CRP synthesis [97,101].

Lactoferrin also affects macrophage polarization, though results are contradictory. Like pentameric CRP, it can induce a shift to M2 macrophages and IL-10 production. Conversely, some studies report lactoferrin-induced pro-inflammatory activity, including IFN-β and IL-6 upregulation in murine macrophages via both TLR4-dependent and -independent pathways [102,103]. Within the TLR4 pathway, LPS binding to CD14 and CD11b/CD18 activates NF-κB and transcription of TNF-α, IL-6, and IL-12 [103]. Other studies show that lactoferrin inhibits LPS binding to CD14, downregulating IL-1, IL-2, and TNF-α production [104,105,106]. Furthermore, several in vitro and in vivo studies demonstrate that lactoferrin decreases IL-6 synthesis [44,107]. In one study, fecal lactoferrin showed sensitivity comparable to calprotectin (0.82 vs. 0.88) for detecting mucosal injury [100]. Levels correlate with endoscopic findings in both UC and CD, though more strongly with UC [108,109]. However, limited stability at room temperature reduces its clinical use, though it remains a promising biomarker for guiding treatment decisions in symptomatic IBD patients [97].

Other biomarkers can modulate macrophage activity. Omentin-1 exerts anti-inflammatory effects by inhibiting TNF-α-induced vascular cell adhesion molecule (VCAM)-1 expression, reducing leukocyte migration [110]. Exosomal miR-223 promotes M2 differentiation, and its absence drives M2 macrophages to produce pro-inflammatory cytokines including TNF-α, IL-1β, and IL-23a [111].

### 2.3. Neutrophils

**Figure 4:**
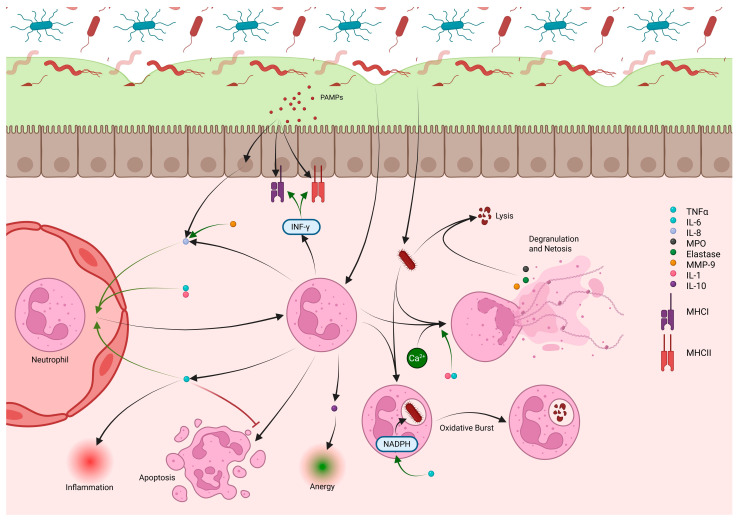
Neutrophils are recruited into the lamina propria from nearby blood vessels under the influence of IL-8, IL-1, and TNF-α. IL-10 produced by recruited neutrophils aids in developing anergy during homeostasis. PAMPs from pathogens cause release of IL-8 from epithelial cells, which, along with IL-6, increases neutrophil recruitment from the circulation. Neutrophils release TNF-α during periods of inflammation as well as additional IL-8 to perpetuate inflammation and recruit more neutrophils. TNF-α prevents apoptosis of neutrophils, prolonging their effect in tissue in addition to recruitment of new neutrophils. INF-γ, also produced by neutrophils, causes increased MHCI and MHCII expression by epithelial cells for better recognition of PAMPs from pathogens. Neutrophils that contact pathogens can phagocytize and destroy bacteria through an oxidative burst fueled by NADPH, which is increased by TNF-α. Neutrophils can use calcium release signaling to degranulate, release cytokines including MPO, MMP-9, and elastase, and undergo netosis. Netosis releases fibrils that can slow and trap bacteria while degranulated neutrophil cytokines (MPO, MMP-9, and elastase) cause lysis of the bacteria. These processes are heightened in the presence of IL-1 and TNF-α. MMP-9 has additional function of improving IL-8 activity in the recruitment of neutrophils.

Recruited neutrophils perform antimicrobial functions aided by inflammatory cytokines. They phagocytose pathogens through PRR binding and generate ROSs via the NADPH pathway, producing oxidative burst activity (Figure 4) [112,113,114]. Pathogens resistant to phagocytosis are targeted through degranulation and neutrophil extracellular traps (NETs), chromatin meshes that immobilize pathogens and enhance exposure to cytotoxic compounds [112,115]. Degranulation releases MPO, elastase, and MMP-9, damaging bacterial membranes and inducing lysis (Figure 4) [112].

Cytokines and chemokines produced by the intestinal epithelium and macrophages, including IL-8, IL-6, CXCL-1, and activated C5a, act as chemoattractants for neutrophils [116]. Neutrophils themselves further amplify recruitment through TNF-α release, which binds tumor necrosis factor receptor (TNFR)1/2 on nearby neutrophils, triggering neutrophil-derived microparticles that increase endothelial adhesion molecules [117]. Cytokines also modulate neutrophil survival and activity. TNF-α extends neutrophil lifespan by activating anti-apoptotic BCL-2 family member A1 (BLF-1), but at higher levels it promotes apoptosis by increasing myeloid leukemia 1 protein (MCL-1) turnover [118]. TNF-α prevents neutrophil egress from inflamed tissues, sustains NADPH-mediated oxidative burst, and enhances MPO expression and calcium signaling, aiding degranulation [114,119]. IL-1 similarly boosts calcium signaling and degranulation [120]. Neutrophils themselves release TNF-α and IL-8 to recruit more neutrophils and macrophages, while MMP-9 potentiates IL-8 activity [112,115]. They also produce IFNγ, which increases MHCI/II expression and supports T-cell activity [121,122], and IL-10, which contributes to inflammatory resolution and wound healing [123].

In IBD, neutrophil activity generally reflects disease progression. In UC, excess neutrophil activity elevates TNF-α, worsening inflammation, barrier permeability, and tissue injury [115]. Increased IL-8 correlates with MPO and ROS-mediated tissue disruption, while elevated MMP-9 enhances neutrophil recruitment [115]. Neutrophils in UC show impaired Intercellular Adhesion Molecule 1 (ICAM-1) suppression, driving recruitment and abscess formation [124], and reduced anti-inflammatory signaling due to decreased lipoxin A4 (LXA4), which normally limits neutrophil migration [115]. NETs also increase thrombotic risk by promoting platelet aggregation [115]. In CD, neutrophil dysfunction is more distinct. Reduced recruitment has been observed, correctable by IL-8 administration, suggesting impaired macrophage-derived IL-8 [115]. Some CD patients exhibit defective respiratory burst activity, impairing bacterial clearance and fostering granuloma formation [5]. Nevertheless, recruited neutrophils still contribute to chronic inflammation and granulation in CD.

Neutrophils release calprotectin, a biomarker secreted primarily by neutrophils but also macrophages and monocytes, which promotes neutrophil recruitment via NF-κB activation [125,126,127]. Calprotectin acts as a damage-associated molecular pattern protein (DAMP) [127], binding the receptor for advanced glycation end products (RAGE) and TLR4 to induce NF-κB signaling cascades that enhance cytokine release (IL-1α, IL-1β, IL-6, IL-8, TNF-α) and adhesion molecule expression (E-selectin, ICAM-1, VCAM-1), thereby facilitating leukocyte transmigration [128,129,130,131,132]. Calprotectin forms S100A8/S100A9 heterodimers that tetramerize in calcium-rich environments, blocking TLR4 binding and self-limiting NF-κB activation [99,100]. Clinically, fecal calprotectin is a well-established biomarker used to distinguish both forms of IBD from IBS, assess disease activity, and predict relapse [125]. It correlates more strongly with UC endoscopic activity than CRP, though inconsistently across studies, and it cannot yet replace colonoscopy [125,133].

Neutrophils also interact with CRP. At inflamed sites, pentameric CRP converts to monomeric CRP, which neutrophils degrade into peptides with anti-inflammatory effects, such as inhibiting neutrophil migration and endothelial adhesion via FC gamma receptor (FcγR)IIIb binding [98,134]. Conversely, CRP binding to FcγRI/IIa receptors can act as an opsonin and further recruit neutrophils to intestinal tissue [135,136].

Like CRP, lactoferrin regulates neutrophil migration. It inhibits LPS-induced expression of E-selectin and ICAM-1 through CD14/LPS binding in the TLR4 pathway [105]. In contrast, MMP-9 promotes recruitment by cleaving IL-8 into a more potent chemokine and generating ProlylGlycineProline (PGP) peptides that signal through C-X-C motif chemokine receptor 2 (CXCR2) [74,137]. IL-8 in turn recruits neutrophils from circulation and induces degranulation, which further enhances MMP activity [74].

### 2.4. Innate Lymphoid Cells

ILCs are a recently described immune population crucial for gut homeostasis and defense against bacterial invasion. ILC1s differentiate under the influence of T-box expressed in T-cells (T-bet) and are characterized by receptors for IL-17, IL-15, and IL-12. Their primary function is production of IFNγ and TNFs following IL-12 stimulation by macrophages [138]. Like neutrophils, ILC1-derived IFNγ enhances MHC function and T-cell activity [121,122].

ILC2s arise under the regulation of retinoic acid-related orphan receptor α (RORα) and GATA3, responding mainly to IL-25 and IL-33 from epithelial cells. IL-25 is secreted in response to commensals, whereas IL-33 is released upon epithelial damage. Both stimulate ILC2 production of IL-13, which drives epithelial proliferation, goblet cell expansion, and barrier repair [138,139]. IL-13 also promotes DC-mediated TH2 differentiation. This is reinforced by IL-4, another ILC2 product induced by IL-25 and IL-33 [140].

ILC3s differentiate under RORγt and AhR signaling [138]. Subtypes include lymphoid tissue inducer (LTi) cells, Natural Cytotoxicity Receptor (NCR)−ILC3s, and NCR+ILC3s. LTi cells support lymphoid organogenesis and produce IL-17A and IL-22. NCR+ILC3s primarily release IL-22, whereas NCR−ILC3s predominantly produce IL-17. IL-22 maintains epithelial homeostasis by inducing mucin and antimicrobial peptide secretion and stimulating IL-18-mediated epithelial proliferation [138]. IL-17 recruits neutrophils and regulates epithelial tight junctions [141]. Upon IL-1β stimulation, ILC3s also produce granulocyte macrophage colony-stimulating factor (GM-CSF), inducing macrophages and DCs to generate RA and IL-10, which support Treg differentiation and tolerance to commensals [141]. Additionally, ILC3s can present commensal antigens on MHCII, dampening T helper responses against microbiota [141].

Altered ILC populations are implicated in IBD. In CD, IL-12, IL-15, and IL-18 drive expansion of IFNγ-producing ILC1s, with elevated IL-12 resulting from increased macrophage activity [138]. Mutations in NOD2 and ATG16L1 further exacerbate pathology: ATG16L1 variants elevate IL-1β, promoting ILC3 differentiation and inflammation [138]. While NCR+ILC3s (producers of IL-17 and IL-22) are reduced in CD, IL-23-responsive NCR−ILC3s are increased, producing excessive IL-17 and IFNγ. Mutations in IL-23R, linked to both CD and UC, contribute to overstimulation and ILC3 apoptosis, suggesting multiple IL-23-mediated mechanisms [142]. Depending on population balance, IL-22 expression may decrease, impairing epithelial repair and bacterial regulation, thereby promoting TH17 differentiation [138,143]. Yet, other studies report increased ILC3-derived IL-22 in both CD and UC, reflecting disease heterogeneity. Excess IL-22 may exacerbate inflammation despite its reparative effects [144]. In UC, elevated IL-33 drives ILC2 expansion and IL-13 production. While normally supportive of epithelial repair, excessive IL-13 increases claudin-2 expression and disrupts barrier integrity [144,145]. In CD, IL-12 can also enhance ILC2 activity and IFNγ production [143,144].

### 2.5. Dendritic Cells

DCs link innate and adaptive immunity through cytokine production and antigen presentation to T-cells. Two major subsets exist: conventional DCs (cDCs), which primarily maintain tolerance in homeostasis and present antigens to T-cells, and the rarer plasmacytoid DCs (pDCs), which secrete type I interferons [146]. cDCs recognize antigens via multiple mechanisms, including uptake of MHCs from other cells, transmembrane transfer from macrophages, direct sampling of the intestinal lumen, and recognition of bacteria–IgA complexes [82,146].

Immature cDCs exposed to commensal antigens without inflammatory co-stimulation are maintained in a tolerogenic state by TGF-β and IL-10. Intestinal epithelial cell-derived RA further induces cDCs to produce RA, strengthening tolerance [146,147]. These RA-producing cDCs migrate to lymph nodes, where they present antigens to T helper cells. By activating latent TGF-β, cDCs drive forkhead box P3 (FOXP3) expression and the differentiation of Tregs and TH17 cells. RA synergizes with TGF-β, favoring Treg over TH17 development, thus promoting tolerance to commensals [147].

When cDC PRRs detect PAMPs in the presence of inflammatory cytokines (e.g., IL-1, TNF-α), cytoskeletal remodeling induces maturation and lymph node migration. Mature cDCs present co-stimulatory molecules such as CD40, driving differentiation of effector T-cells [148]. PRR signaling typically induces IL-12 and IFNγ, which promote TH1 differentiation while suppressing TH2 responses [149]. Inflammatory cytokines may also suppress p38 MAPK, reducing RA production and shifting differentiation from Tregs to TH1 cells [146]. Alternatively, PRR signaling that promotes TH2 differentiation induces epithelial IL-25 and IL-33, which suppress IL-12 in cDCs and instead stimulate CCL7 secretion. CCL7 recruits basophils, which collaborate with cDCs to produce IL-4, initiating TH2 differentiation [146,148,150]. Certain Gram-negative PAMPs also trigger cDC IL-23 secretion, favoring TH17 differentiation [148].

Defects in DC function parallel those of macrophages in IBD. Mutations in NOD2 and ATG16L impair autophagy, reducing bacterial sensing, phagocytosis, and clearance. Excess inflammatory cytokines enhance DC activity and T-cell stimulation, as reflected by elevated CD40 expression in inflamed CD tissue [151,152,153]. Normally, epithelial TSLP suppresses DC activity, but this regulation is diminished in IBD, leading to increased IL-12 production [153].

A central role of DCs in IBD is loss of tolerance to commensals. One study found circulating DCs in IBD patients displayed increased programmed death ligand (PD-L)2 aggregation, which interfered with the PD-L1–programmed death receptor (PD-1) interactions required for maintaining T-cell anergy. Disruption of this pathway enhanced inflammation and tissue damage [154]. In UC, CD103+ DCs failed to induce Tregs, instead producing IFNγ and IL-17, thus amplifying inflammation. This dysfunction was associated with elevated TLR2 and TLR4 expression [155]. Normally, the exosomal miR-223 downregulates differentiation of pro-inflammatory CD103+ DCs. Loss of miR-223 permits DC-driven secretion of IL-23, IL-12, TNF-α, and IL-6. Mice deficient in miR-223 exhibited pro-inflammatory CD103+ DCs and developed colitis [111,156].

### 2.6. T-Cells

**Figure 5:**
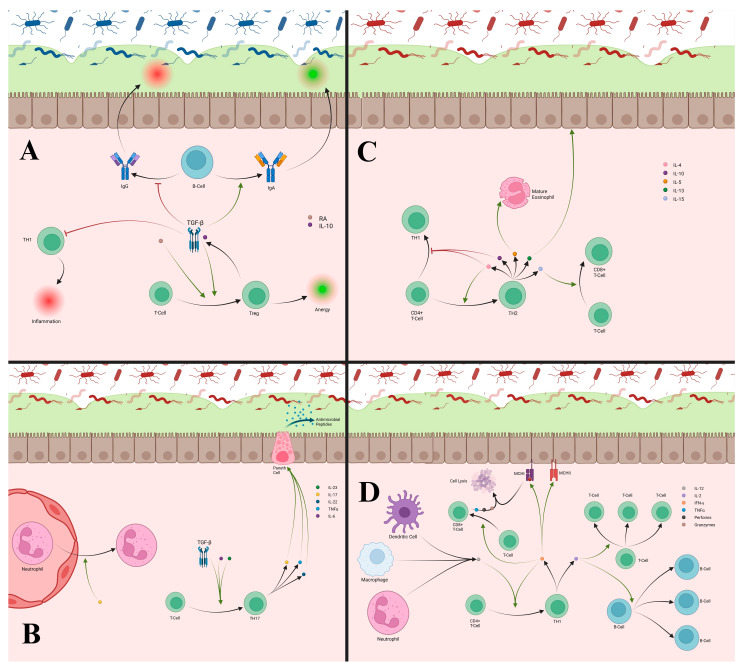
(**A**) T-cells differentiate into Treg cells under the influence of TGF-β or RA. These Tregs promote anergy and release additional TGF-β to perpetuate Treg differentiation. TGF-β inhibits T-cell differentiation into TH1 cells, promotes IgA production from B-cells, and decreases IgG production, which all contribute to reducing inflammatory responses. (**B**) T-cells differentiate into TH17 cells with the combined influence of TGF-β, IL-6, and IL-23. TH17 cells release IL-17, Il-22, and TNF-α, which all stimulate Paneth cells to improve anti-microbial peptide production and help with defense against invading pathogens. IL-17 also aids in recruitment of neutrophils into the lamina propria, which can increase inflammation. (**C**) TH2 cells differentiate from CD4+ T-cells under the influence of IL-4 and produce several cytokines including IL-4, IL-10, IL-5, IL-13, and IL-15. IL-4 and IL-10 produced by TH2 cells both reduce the differentiation of TH1 cells, helping attenuate inflammatory responses. IL-4 production from TH2 cells can also create a positive feedback loop while suppressing TH1 cells. IL-5 produced by TH2 cells is important for proper eosinophil maturation, which is important for defense against parasites. IL-13 helps repair the epithelial barrier that can be damaged by inflammation. IL-15 influences the differentiation of T-cells into cytotoxic CD8+ T-cells, though TH2 cells are not a major source of this cytokine. (**D**) TH1 cells differentiate from T-cells when exposed to IFNγ and IL-12 and will release IFNγ and IL-2. IFNγ aids in T-cell differentiation into cytotoxic CD8+ T-cells and increases the number of MHCI and MHCII receptors, improving recognition of PAMPs and their downstream signaling. The cytotoxic T-cells recognize MCHI receptors bound to PAMPs to trigger lysis of the attached cell to eliminate intracellular pathogens through the release of perforins, granzymes, and TNF-α. IFNγ from TH1 cells also crease a positive feedback loop for TH1 differentiation. IL-2 aids in the proliferation of T-cells and B-cells to further maintain immune responses.

T-cells encompass multiple subtypes with distinct roles in immunity. In homeostasis, Tregs dominate to maintain tolerance to commensals and suppress inflammation. Differentiation is largely driven by TGF-β, which alone induces Tregs and TH17 cells, with RA from DCs further supporting this process (Figure 5) [157]. Tregs secrete IL-10 and TGF-β to attenuate inflammation; TGF-β additionally suppresses effector T-cell function, reduces IgG, and enhances IgA production by B-cells, reinforcing barrier homeostasis (Figure 5) [158]. This IgA coats commensals to modulate antigenicity, lowering immune activation, yet can still aid immunity via binding to bacterial antigens and M-cells in Peyer’s patches [159].

TH17 cells arise when TGF-β is combined with IL-6 and IL-23, producing IL-17A, IL-17F, IL-22, and TNF-α (Figure 5) [138,160]. IL-17 and TNF-α stimulate antimicrobial peptide and cytokine production by epithelial cells, recruit neutrophils via CXCL5, and increase epithelial permeability for neutrophil migration [12,15,16,161]. IL-22 supports barrier defense by promoting antimicrobial peptides and epithelial proliferation [160].

TH1 cells, which produce IL-2 and IFNγ, differentiate in the presence of IFNγ and, aided by IL-12 from antigen-presenting cells (APCs), create feedback loops that drive TH1 polarization (Figure 5) [162]. IFNγ enhances antigen presentation by upregulating MHC-I and MHC-II and supports CD8+ T-cell differentiation [122]. CD8+ T-cells mediate cytolysis by releasing perforins and granzymes, which activate caspases and induce apoptosis. CD8+ T-cells also secrete IFNγ and TNF-α, enabling clearance of intracellular pathogens and parasites resistant to phagocytosis [163,164]. IL-2 further promotes clonal expansion of B- and T-cells (Figure 5) [165].

TH2 cells exhibit mixed pro- and anti-inflammatory activity, particularly in parasitic defense. They produce IL-4, IL-5, IL-10, IL-13, and IL-15 (Figure 5). IL-4 drives TH2 differentiation and self-propagation [166], while IL-4 and IL-10 counterbalance TH1-driven inflammation [165,167]. IL-5 promotes eosinophil maturation [168], IL-13 supports epithelial repair [138], and IL-15 facilitates CD8+ T-cell differentiation and survival [163,169].

T follicular helper (Tfh) cells (PD-1+ ICOS+) localize in B-cell follicles and regulate germinal center formation when activated by DCs. Through Inducible Co-Stimulator (ICOS), Tfh cells interact with B-cells, promoting selection of high-affinity clones, plasma cell differentiation, and Ig class switching. Their cytokines, IL-21 and IL-4, drive IgG and IgA switching, though IL-4 favors IgG and limits IgA despite IL-21 signaling [170,171,172,173,174].

**Figure 6:**
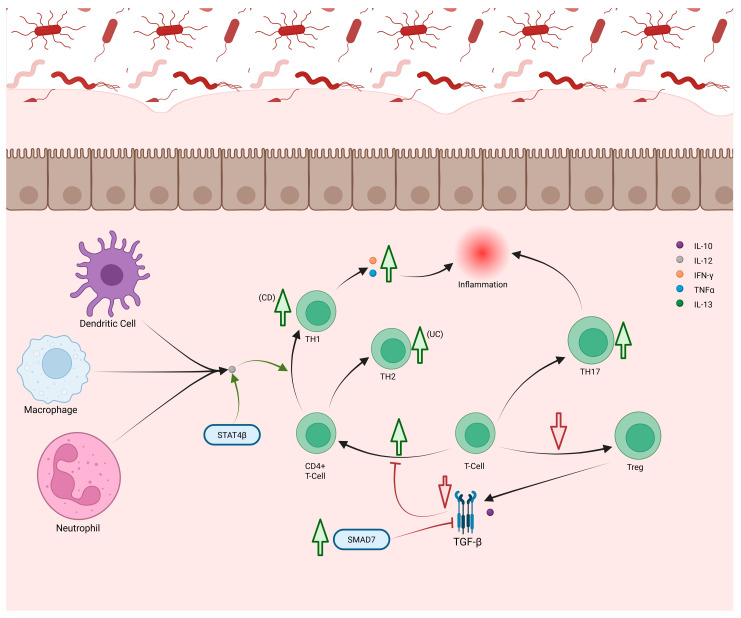
TH1 and TH2 cells are both increased in CD and UC, though in different proportions. Higher levels of TH1 are seen in CD, while more TH2 cells are seen in UC. Increase in TH1 cells leads to increased levels of TNF-α and IFNγ production, worsening inflammation. Mutations in STAT4β gene potentiates IL-12 produced by other immune cells, improving TH1 differentiation. Mutations in SMAD7 weakens the activity of TFG-β, normally an inhibitor of CD4+ T-cell differentiation. The loss of this inhibition causes increased TH1 and TH2 differentiation. De-creased differentiation of T-cells to Tregs also diminishes the production of TGF-β and IL-10 leading to loss of anti-inflammatory signaling. Increased differentiation of Th17 has also been noted, contributing to increased inflammation through neutrophil recruitment.

In IBD, increased effector CD4+ T-cells and reduced Tregs drive inflammation (Figure 6) [175]. Elevated CD4+ T-cells correlate with active disease and normalize in remission [176]. Excess IL-12 from APCs promotes TH1 differentiation through signal transducers and activators of transcription (STAT)4, whose β isoforms are upregulated in IBD, enhancing TNF-α expression [177]. Single-nucleotide polymorphisms (SNPs) in the IFN and TNF genes further elevate IFNγ and TNF-α [177]. Both CD and UC show increased TH1 cells, though CD exhibits a higher TH1/TH2 ratio, while UC skews toward TH2 (Figure 6) [177]. In UC, excess IL-13 from TH2 and ILC2 cells disrupts epithelial barrier integrity [144,145,177]. Type 1 cytotoxic CD8+ T-cells (Tc1) producing IFNγ and TNF-α are also elevated in both CD and UC [125]. Tregs mitigate inflammation via TGF-β and IL-10, but their numbers are reduced during active IBD and recover in remission [178]. In CD, SMAD7 overexpression inhibits TGF-β signaling, impairing Treg function (Figure 6) [178].

Although TH17 cells are elevated in CD, anti-IL-17A therapy worsens disease, consistent with IL-17A’s protective role in limiting TH1 differentiation. Thus, increased TH17 activity may represent a compensatory response to chronic TH1 inflammation [177]. However, IL-17A also contributes to pathology by recruiting granulocytes and stimulating inflammatory cytokines from epithelium and macrophages [179]. Microbiome changes during active IBD can further skew Treg/TH17 balance, reducing tolerance and heightening inflammation (Figure 6) [180].

T-cell-related biomarkers also influence inflammation. NO reduces TH1 activity by suppressing IL-12 synthesis in macrophages and DCs, prevents nitrate binding of the RORγt promotor region of IL-17 gene inhibiting IL-17 transcription, and promotes Treg induction [181,182]. In contrast, lactoferrin enhances TH1 responses by upregulating IL-12 and CD40 via TLR4 [103,183]. TH2-derived IL-13 induces IL-6 through STAT6, which in turn induces omentin-1, while TNF-α both increases IL-6 and suppresses omentin-1. This TNF-α/IL-6 ratio may regulate omentin-1 expression, whose levels are reduced in UC and CD [184].

### 2.7. B-Cells

After activation by APCs, T helper cells bind antigen-specific B-cells to induce immunoglobulin production. In some cases, B-cells in GALT recognize antigens directly through TLR and B-cell receptors, enabling T-cell-independent responses. In homeostasis, these B-cells differentiate into plasma cells producing secretory IgA (sIgA), the primary antibody for surveillance against foreign antigens. Class switching to antigen-specific IgA occurs through several mechanisms. APCs phagocytize and present commensal bacterial antigens to T helper cells, which then activate B-cells via CD40/CD40 ligand binding [185]. DCs enhance this process by producing TGF-β-induced A proliferation-inducing ligand (APRIL) and B-cell activating factor (BAFF), promoting plasma cell differentiation and IgA class switching [185,186,187]. Activated B-cells may become short-lived plasma cells or migrate to follicles to form germinal center (GC) reactions with Tfh cells and DCs [185,186]. IL-21 from Tfh cells drives IgA or IgG class switching, though IgA switching occurs only in the absence of IL-4. Antigen-specific IgG is produced through similar T-cell-dependent mechanisms, with IFNγ and IL-17 influencing class switching. IL-21, particularly in the presence of IL-4, promotes IgG switching [185]. Polysaccharide antigens can induce IgG switching through direct binding to B-cell TLRs [185]. IgG binds FcγR to regulate immune responses. In macrophages, this interaction increases cytokine production; in neutrophils, it enhances antimicrobial activity; and in DCs, it promotes phagocytosis and MHCII expression [185,188]. FcγRIIb binding on B-cells suppresses antibody production, creating a negative feedback loop [188].

Altered B-cell populations and immunoglobulin profiles have been observed in IBD. In active CD, IgM memory B-cells are reduced, while IgG and IgA class-switched B-cells are increased. This impaired B-cell maturation resolves with anti-TNF-α therapy. Though the underlying cause for this resolution remains unclear, reduced microbial exposure has been proposed in modern environments [189,190]. This reduction in CD27+ IgM memory cells is unique to CD and helps distinguish it from UC [190]. In CD, IgG and IgA B-cells accumulate around granulomas and may contribute to their formation by impairing bacterial clearance [190]. Elevated IgG and IgA, including anti-Saccharomyces cerevisiae antibodies (ASCAs), are strongly associated with CD, and some autoantibodies, such as anti-flagellin, can appear years before diagnosis [191]. In UC, anti-tropomyosin 5 (anti-TM5) and anti-epithelial autoantibodies are implicated in disease progression [192]. Chronic UC inflammation alters IgG and IgA responses in GCs, with decreased variable, diversity, joining (VDJ) mutation and impaired GC maturation, potentially expanding autoreactive B-cell populations. Increased inflammation also recruits autoreactive plasma cells and depletes IL-10-producing regulatory B-cells (Bregs) [193,194].

Both UC and CD show elevated IgG against microbiota, indicating loss of tolerance and chronic inflammation [191]. Excessive TH1 activity further drives IgG class switching, amplifying inflammation [191,194]. Structural changes in IgG, including increased agalactosylation and decreased sialylation, enhance pro-inflammatory signaling in both diseases, with reduced IgG sialylation particularly noted in CD [191].

## 3. Biomarker Challenges and Future Directions

Biomarkers in IBD have been studied for their usefulness in diagnosis, prognostic indication, and disease management. A prominent example of the utility of biomarkers follows the STRIDE II treat-to-target strategies most recently updated in 2021 [195]. Currently, biomarkers such as CRP and fecal calprotectin (FC) can help guide smaller changes in treatment strategies; however, while more invasive, endoscopic and biopsy confirmation is still needed to document and direct major treatment changes. Newer initiatives have stressed the importance of moving from these invasive means of IBD treatment management to pursuing laboratory testing, or ideally point of care (POC)-friendly modalities that fulfill the ASSURED (affordable, sensitive, selective, user-friendly, rapid, and robust, equipment-free, deliverable) goals of POC testing [196]. POC testing would ideally be highly sensitive to detecting inflammation in the gut and quantifiably correlated with increased disease burden [196]. As discussed previously, better understanding of the cytokine pathways involved in IBD pathology can help better understand how to use clinical biomarkers and aid in discovery of new biomarkers.

Serum CRP in IBD is heavily associated with M1 activity and heavily influences production of IL-6, IL-1β, and TNF-α [93]. Because of this association with macrophages and IL-6, CRP is more strongly correlated with CD than it is with UC [95,96]. In CD, elevated high sensitivity CRP has an extremely high sensitivity as high as 70–100% compared to only 50–60% sensitivity in UC [98,197]. Despite these observations, CRP is still currently used to identify IBD and monitor active disease activity, but not necessarily to distinguish CD from UC due to wide range of CRP thresholds used [198,199]. Because CRP is present in many inflammatory diseases, it is a useful tool in identifying active disease but may not be effective in differentiation of IBD specifically. Erythrocyte sedimentation rate is another common biomarker associated with non-specific inflammation and is not necessarily directly related to a specific cytokine. It has a sensitivity of 66% and specificity of 84% for the diagnosis IBD and thus is less helpful for diagnosis compared to CRP, particularly when differentiating IBD from IBS, though in combination can still help with serologic testing (Table 1) [196,198,199].

ASCA and perinuclear anti-neutrophil cytoplasmic antibodies (p-ANCAs) were discovered in the 1990s with association with CD and UC, respectively [196]. When these biomarkers are combined in an assay to differentiate between CD and UC, ASCA+/p-ANCA- has a diagnostic sensitivity of 50.7% and specificity of 80.5% for CD. For UC, ASCA-/p-ANCA+ has a diagnostic sensitivity of 31.7% and specificity of 94.4% [196,198,200]. ASCA has been more strongly associated with ileal forms of CD from other colonic forms [201]. It has no strong connection with cytokines; however, it is suspected that ASCA activity correlates with TH1 activity. This has been associated with elevated levels of TNF-α and IL-12, which are believed to be due to anti-yeast activity [202]. At this time, P-ANCA has no consistent connection to cytokine pathways found in UC (Table 1). Though these biomarkers help with differentiation and diagnosis, seronegative forms of CD and UC exist, limiting their usefulness.

While previously discussed in the context of intestinal epithelial cells, NO also decreases IL-8 production, limiting leukocyte recruitment [203]. Cytokines like TNF-α, IL-1α, and IFNγ from colonic epithelial cells increase NO production, while TH2-related IL-4 and IL-13 decrease production [203]. This reduction accounts for its heavy association with UC activity by favoring TH2 differentiation, in addition to suppression of IL-12 to prevent M1 differentiation [204]. NO has shown significant promise in differentiating between active and inactive UC with 100% sensitivity and specificity for a cutoff of 17.4 μM and 88% sensitivity and 69% specificity for CD with a cutoff of 14.0 μM [196,203]. Though the use of NO is promising for disease monitoring of UC, most data have been collected from induced animal models rather than human disease studies [203]. There have been few studies to standardize the means of collection or thresholds for usage of NO, further limiting its usage (Table 1) [59,205].

Omentin-1 exerts anti-inflammatory effects on several cell types and is decreased in both forms of IBD. While it does not interfere directly with TNF-α, it does interfere with its recruitment of macrophages as previously discussed. TNF-α, however, can lead to omentin-1 suppression through IL-6 suppression [110,184]. While decreases are seen in both UC and CD, sensitivity and specificity for detection of disease activity were slightly better in CD, showing a sensitivity of 94.9% and specificity of 88.7%, compared to 72.4% and 82%, respectively. A key limitation in the use of omentin-1 for IBD is the lack of standardization and correlation with endoscopic disease activity. Omentin-1 is also decreased in obesity, which can be a confounding factor in appropriate interpretation (Table 1) [206,207].

While miR-223 has mostly anti-inflammatory functions, it can worsen inflammation in some cases by weakening epithelial barriers in response to IL-23 and IL-17 [48]. When present, miR-223 suppresses production of TNF-α, IL-1β, IL-6, and IL-23a from M2 and DC cells to limit inflammation [111,208]. miR-223 is a promising, non-invasive method for evaluation of IBD, though appropriate cutoffs, usage, and collection have not been standardized [209,210]. The sensitivity and specificities of using miR-223 have not been well established and require more study (Table 1).

FC and fecal lactoferrin are common fecal biomarkers used to diagnose and monitor IBD. FC is primarily associated with neutrophil and macrophage activity and the production of IL-1α, IL-1β, IL-6, IL-8, TNF-α [128,129,130,131,132]. FC is more sensitive to predicting endoscopic inflammatory activity in UC than CRP or erythrocyte sedimentation rate (ESR) and is associated with 83% sensitivity and 74% specificity (cutoff ≤ 168 μg/g) for predicting a sustained clinical response at 1 year as well as a 79% sensitivity and a 57% specificity (cutoff ≤ 121 μg/g) for predicting endoscopic healing in UC [195]. FC also is useful in understanding endoscopic disease activity in CD, with a sensitivity of 82% and specificity of 72% [198,211]. Related to calprotectin is S100A12, a subunit of calprotectin, has shown a sensitivity of 96% and specificity of 92% for IBD [196,212,213]. Fecal lactoferrin is associated with multiple cell types, with often conflicting effects on cytokine production [102,103,104,105,106]. Lactoferrin has shown significant sensitivity and specificity to CD and UC, and it correlates well with endoscopic activity [196,198,214]. A significant limitation is the large variety of cutoffs and usage, making accurate application of FC and lactoferrin difficult due to the lack of standardization (Table 1).

MPO correlates closely with other biomarkers including fecal calprotectin and lactoferrin and may be useful as a non-invasive biomarker [196]. It is typically associated with elevated IL-8 and TNF-α, which increase neutrophil activity and MPO release [115]. On its own, MPO had a sensitivity of 74% and specificity of 84%, though sensitivity increased to 82% when used with fecal calprotectin to diagnose IBD [215]. MMP-9 is also produced by neutrophils and is affected by IL-8 and TNF-α. It also potentiates IL-8 activity creating a feedback loop [112,115]. Fecal MMP-9 has been shown to have a high sensitivity of 96% for clinical and endoscopic activity of UC and a specificity of 75% [196]. It also has high sensitivity for CD, though specificity is low [84]. This is likely because MMP-9 fecal activity better informs activity of colonic disease, which is more useful in UC and colonic CD but misses many other CD patients (Table 1) [216].

The biomarkers discussed can have a wide range of uses including diagnosis, severity assessment, and response to treatment. Predicting treatment response prior to initiation is being investigated with many biomarkers; however, none have been conclusive so far except CRP, which could be correlated to the likelihood of response to anti-TNF treatment in CD due to significant involvement of neutrophils and TNF-α production [217]. ASCA has been studied for treatment response without success, but it is a useful tool for diagnosis and predicting severity, with higher ASCA in CD correlated with more severe stenosis and need for surgery [218]. p-ANCA similarly correlates with UC disease severity rather than guidance in treatment [219]. Serum and fecal miR-223 have shown significant connection to disease severity and can be used to monitor treatment success, but they have not shown any ability to predict success prior to treatment initiation [220]. Fecal calprotectin is very helpful in diagnosing, monitoring for treatment response, and monitoring of relapse but has not been associated with response prediction [221].

The lack of validation and standardized guidelines for the use of biomarkers in the diagnosis and treatment of IBD limits the clinical significance of their use at this time. There is still more research needed to determine how these biomarkers and cytokines might be integrated into the screening, diagnosis, and surveillance of UC and CD. Currently, endoscopic biopsies remain the most crucial component for the diagnosis and observation of IBDs. However, the prospect for use of biomarkers at POC has tremendous potential for reducing disease burden in the CD and UC population.

## 4. Treatment of IBD

The treatment of IBD relies on a broad spectrum of strategies, ranging from lifestyle modification and pharmacological therapy to surgery in severe or refractory cases. Because IBD is a chronic disease characterized by periods of remission and flare, therapeutic success is measured not only by the ability to induce but also maintain remission.

Aminosalicylates such as sulfasalazine and mesalazine are first-line agents, particularly in UC. Therapeutic effect is mediated by 5-aminosalicylic acid (5-ASA), which reduces prostaglandin production from arachidonic acid, scavenges ROS, and dampens pro-inflammatory cytokine release. A key mechanism involves inhibition of ROS-driven NF-κB activation, thereby decreasing expression of TNF-α and IL-6. In parallel, 5-ASA interferes with JNK and p38 signaling pathways, further reducing levels of IL-6, IL-1β, and TNF-α. Importantly, it can also promote Treg differentiation and enhance TGF-β through AhR activation. While effective in both UC and CD, the role of 5-ASA in CD maintenance remains debated. Side effects are often mild, though intolerance develops in approximately 15% of patients, and rare severe complications such as pancreatitis or nephrotoxicity may occur [222,223,224,225,226,227].

Corticosteroids remain indispensable for managing acute flares. Their action is mediated by glucocorticoid receptor (GR) binding, which suppresses NF-κB and activator protein 1 (AP-1) activity and prevents induction of TNF-α, IL-1β, and IL-6. Corticosteroids also enhance MKP-1, which blocks JNK and p38 phosphorylation, thereby destabilizing mRNA transcripts for TNF-α, IL-6, and IL-8. Beyond cytokine modulation, they strengthen the epithelial barrier by inhibiting myosin light chain kinase (MLCK) and STAT1 pathways triggered by TNF-α, IFNγ, and IL-1β, while also promoting IL-10 production. Their efficacy in both UC and CD is clear, though long-term use is limited by systemic side effects. Targeted formulations such as budesonide offer more localized intestinal delivery with improved tolerability [222,227,228,229,230,231,232].

For maintenance of remission, immunomodulators such as thiopurines play a critical role. Azathioprine, 6-mercaptopurine, and 6-thioguanine are metabolized into deoxy-6-thioguanosine phosphate (deoxy-6-TGNP), which incorporates into DNA and disrupts T-cell replication, while also binding RAC1 to inhibit B- and T-cell proliferation. Despite their usefulness in both UC and CD, adverse events such as leukopenia, hepatotoxicity, and flu-like symptoms limit their tolerability in some patients [222,227,233].

A major advance in IBD management came with the introduction of biologics targeting specific cytokines. Anti-TNF therapies—including infliximab, adalimumab, and golimumab—are highly effective in reducing disease activity, particularly in CD, and in lowering colectomy rates in UC. Nevertheless, up to 40% of patients fail to respond to initial therapy, and many lose responsiveness over time, often due to immunogenicity or alternative inflammatory pathways. More recently, attention has shifted to the IL-12/23 axis. Ustekinumab, an antibody targeting the shared p40 subunit of IL-12 and IL-23, has shown efficacy in both UC and CD, while newer agents directed specifically against IL-23, such as mirikizumab and risankizumab, are emerging with strong early results [222,227,234]. SCFA has shown promising data along with CRP regarding use as a predictive therapeutic biomarker for anti-TNF therapies [217].

Another pathway involves leukocyte trafficking, where anti-integrin therapies have provided an additional layer of targeted intervention. Vedolizumab, which blocks α4β7 binding to MAdCAM-1, prevents gut-homing of lymphocytes and has proven effective for both induction and maintenance of remission with an excellent safety profile. Etrolizumab, which targets β7 integrins, and carotegrast methyl, which targets α4, are newer agents under investigation, particularly for UC [222].

In parallel, small molecule drugs have gained momentum as convenient oral alternatives. Janus kinase (JAK) inhibitors such as tofacitinib and upadacitinib prevent cytokine-mediated activation of the JAK-STAT pathway, particularly downstream of IFNγ. Tofacitinib is now well established in UC, while upadacitinib shows benefit in both UC and CD. Other intracellular targets include sphingosine 1-phosphate receptor (S1PR) modulators such as ozanimod, which prevent lymphocyte egress from lymph nodes and have demonstrated efficacy in UC. Phosphodiesterase 4 (PDE4) inhibitors such as apremilast act by stabilizing cyclic adenosine monophosphate (cAMP) and guanosine monophosphate (cGMP) to reduce NF-κB-driven cytokine expression, though clinical benefit has been more modest. Novel approaches such as cobitolimod, a TLR-9 agonist that boosts IL-10 production, also hold promise in UC [235,236].

Beyond pharmacological interventions, manipulation of the gut microbiota has been explored through fecal microbiota transplant (FMT). By restoring commensal balance and increasing SCFA production, FMT promotes anti-inflammatory cytokine release, tolerance, and epithelial barrier integrity. Clinical trials suggest therapeutic benefit in UC and CD, though practical and safety considerations still limit its broader use [222,237,238].

Looking ahead, the therapeutic landscape continues to evolve. Combination therapies are being actively investigated, while novel strategies such as chimeric antigen receptor T-cell (CAR-T) cells engineered against CD19+ B-cells represent a frontier in precision immunotherapy. Early studies indicate that these CAR-T cells may achieve deeper and more durable B-cell depletion than rituximab, highlighting the potential for transformative change in IBD treatment [239].

## 5. Conclusions

IBD is a complicated, heterogeneous disease with a wide variety of areas for disruption of the natural protective systems in the GI tract. A better understanding of the associated immunological systems, cytokine pathways, and biomarkers help guide future research and clinical application of new treatments. The discovery and application of new biomarkers serve as a valuable opportunity to non-invasive diagnostic tools not reliant on endoscopic evaluation. Application of these biomarkers to predictive therapeutic strategies will also significantly help direct the ever-growing clinical management as new and more effective treatments for both UC and CD are developed.

## Figures and Tables

**Table 1:** List of discussed biomarkers with associated uses, sensitivities, specificities, and major barriers divided by serologic versus fecal collection and by use in Crohn’s disease versus ulcerative colitis. * designates biomarkers that do not distinguish between CRP and ESR.

Crohn’s Disease					Ulcerative Colitis			
Biomarker	Use	Sensitivity	Specificity	Cytokine Association	Use	Sensitivity	Specificity	Cytokine Association
**Serologic Biomarkers**								
CRP *	Endoscopic disease activity	49%	92%	IL-6, IL-1β, TNF-α	**-**	**-**	**-**	**-**
	Diagnosis	63%	88%					
**ASCA/** **p-ANCA**	Diagnosis (CD vs. UC) (ASCA+/p-ANCA-)	50.7%	80.5%	TNF-α, IL-12	Diagnosis (UC vs. CD) (ASCA-/p-ANCA+)	31.7%	94.4%	**-**
**High** **Sensitivity CRP**	Endoscopic disease activity	70%	**-**	IL-6, IL-1β, TNF-α	Endoscopic disease activity	50%	**-**	IL-6, IL-1β, TNF-α
ESR *	Diagnosis	66%	84%	**-**	**-**	**-**	**-**	**-**
**MiR-223**	**-**	**-**	**-**	IL-23, IL-17, TNF-α, IL-1β, IL-6	**-**	**-**	**-**	IL-23, IL-17, TNF-α, IL-1β, IL-6
**Omentin-1**	Disease activity	94.9%	88.7%	TNF-α, IL-6	Disease activity	72.4%	82%	TNF-α, IL-6
**Fecal Biomarkers**								
**Calprotectin**	Diagnosis *	88%	80%	IL-1α, IL-1β, IL-6, IL-8, TNF-α	Diagnosis	**-**	**-**	IL-1α, IL-1β, IL-6, IL-8, TNF-α
	Endoscopic disease activity	82%	72%		Endoscopic disease activity	87%	77%	
					Treatment response	83%	74%	
					Endoscopic healing	79%	57%	
**Lactoferrin**	Diagnosis	75%	100%	IL-6, TNF-α, IL-12, IL-10	Diagnosis	82%	100%	IL-6, TNF-α, IL-12, IL-10
	Endoscopic disease activity	82%	71%		Endoscopic disease activity	81%	82%	
MPO *	Diagnosis	74.7%	84.6%	IL-8, TNF-α	**-**	**-**	**-**	**-**
**MMP-9**	Endoscopic disease Activity	90%	63.6%	IL-8, TNF-α	Endoscopic disease activity	96%	75%	IL-8, TNF-α
**MiR-223**	**-**	**-**	**-**	IL-23, IL-17, TNF-α, IL-1β, IL-6	Diagnosis	86.7%	90%	IL-23, IL-17, TNF-α, IL-1β, IL-6
					Prediction of Treatment success	25%	92.5%	
**NO**	Endoscopic disease activity	88%	69%	IL-12, IL-8, TNF-α, IL-1α, IFNγ, IL-4, IL-13	Endoscopic disease activity	100%	100%	IL-12, IL-8, TNF-α, IL-1α, IFNγ, IL-4, IL-13
**S100A12**	Diagnosis	96%	92%	**-**	Diagnosis	91%	96%	**-**

## Data Availability

No new data were created or analyzed in this study. Data sharing is not applicable to this article.

## Abbreviations

The following abbreviations are used in this manuscript:

5-ASA	5-Aminosalicylic Acid
AhR	Aryl Hydrocarbon Receptor
anti-TM5	Anti-Tropomyosin 5
AP-1	Activator Protein 1
APCs	Antigen-Presenting Cells
APRIL	A Proliferation-Inducing Ligand
ASCA	Anti-Saccharomyces Cerevisiae Antibodies
ATG	Autophagy-Related Gene
ATG16L1	Autophagy-Related 16-Like 1
ATOH1	Atonal Homolog 1
ATP	Adenosine Triphosphate
BAFF	B-Cell-Activating Factor
BCL-2	B-Cell Lymphoma 2
BLF-1	BCL-2 Family Member A1
Breg	Regulatory B-Cell
cAMP	Cyclic Adenosine Monophosphate
CAR-T	Chimeric Antigen Receptor T-Cell
CCL2/CCL7	Chemokine (C-C Motif) Ligand 2 and 7
CCR2	C-C Motif Chemokine Receptor 2
CD	Crohn’s Disease
cDCs	Conventional DCs
cGMP	Cyclic Guanosine Monophosphate
COSMC	Core 1 β3-Galactosyltransferase-Specific Molecular Chaperone
CRP	C-Reactive Protein
CXCL	C-X-C Motif Chemokine
CXCR2	C-X-C Motif Chemokine Receptor 2
DAMP	Damage-Associated Molecular Pattern Protein
M1	Macrophage Type 1
M2	Macrophage Type 2
MAdCAM	Mucosal Vascular Addressin Cell Adhesion Molecule
MAPK	Mitogen-Activated Protein Kinase
MCL-1	Myeloid Leukemia 1
MCP-1	Monocyte Chemoattractant Protein 1
MHC	Major Histocompatibility Complexes
MiR	MicroRNA
MLCK	Myosin Light Chain Kinase
MMP-9	Matrix Metalloproteinase 9
MPO	Myeloperoxidase
NADPH	Nicotinamide Adenine Dinucleotide Phosphate
NETs	Neutrophil Extracellular Traps
NCR	Natural Cytotoxicity Receptor
NK1R	Neurokinin-1 Receptor
NF-κB	Nuclear Factor Kappa-Light-Chain-Enhancer of Activated B-Cells
NOD2	Nucleotide-Binding Oligomerization Domain-Containing Protein 2
NO	Nitric Oxide
NOX1	NADPH Oxidase 1
PAMPs	Pathogen-Associated Molecular Patterns
p-ANCA	Perinuclear Anti-Neutrophil Cytoplasmic Antibodies
PARS	Poly(ADP-ribose) Synthetase
PDE4	Phosphodiesterase 4
PD-1	Programmed Death Receptor 1
PD-L	Programmed Death Ligand
pDCs	Plasmacytoid DCs
PGP	ProlylGlycineProline
